# Comparative Large Amplitude Oscillatory Shear (LAOS) Study of Ionically and Physically Crosslinked Hydrogels

**DOI:** 10.3390/polym15061558

**Published:** 2023-03-21

**Authors:** Thomas B. Goudoulas, Anna Didonaki, Sharadwata Pan, Ehsan Fattahi, Thomas Becker

**Affiliations:** Chair of Brewing and Beverage Technology, TUM School of Life Sciences, Technical University of Munich, Weihenstephaner Steig 20, 85354 Freising, Germany

**Keywords:** alginate, gelatin, hydrogel, physically crosslinking, ionically crosslinking, *ex situ* gelation, yielding, large amplitude oscillatory shear (LAOS), shear stress decomposition, MITlaos

## Abstract

Hydrogels are highly versatile and widely applicable materials within various scientific, technological, and food sectors. Alginate and gelatin hydrogels, along with their crafted variations, are possibly the most common ones. However, the ionic crosslinking of alginate-Ca^++^ is a different gelation mechanism than the physical crosslinking of gelatin. In this work, we prepare alginate-Ca^++^ hydrogels using individual layer gelation and experimentally evaluate LAOS rheological behavior. We apply shear-stress decomposition using the MITlaos software and obtain the elastic and viscous contributions within the nonlinear response of the individual alginate-Ca^++^ layer. We compare these results with the nonlinear responses of the gelatin-alginate *ex situ* individual layer. The strain-sweep patterns are similar, with loss modulus overshoot. The applied shear can destroy the larger-scale structural units (agglomerate/aggregates), resulting in analogous patterns. However, the critical strain points are different. Based on the shear-thickening ratio T of the LAOS analysis, it can be assumed that the common feature of *ex situ* preparation, i.e., gelation as individual layers, provides a matching bulk microstructure, as the hydrogels differ significantly at a molecular-binding level.

## 1. Introduction

Hydrogels and hydrogel-based materials for various food and biomedicine applications have attracted increasing attention due to the outstanding biocompatibility of the basic compounds, ease of preparation, and versatility in tuning their mechanical properties.

Polysaccharides are widely used biocompatible and versatile biopolymers, and alginates are possibly the most applicable member of this group of compounds [1,2]. Alginates are linear copolymers composed of (1–4) linked *b*-D-mannuronic acid (usually called the M-block) and *a*-L-guluronic acid (the G-block), mainly extracted from brown seaweed as natural polysaccharides. It is established that the Ca^++^ ions favorably bind to G-blocks rather than to M-blocks [3,4]. Consequently, the mannuronic-to-guluronic ratio (G/M ratio) appears to significantly impact crosslinking between cations and the gel structure, along with the molecular weight (Mw) and the biopolymer concentration [4]. Therefore, the alginate-Ca^++^ gels are characterized by ionic crosslinking junction zones that hold the alginate chains together [1,3]. For a given alginate concentration, the influence of the Ca^++^ concentration on the strength of the developed gel is of critical importance [5]. Cuomo et al. [3] evaluated the mechanical strength of hydrogels prepared at 1 wt.% alginate by comparing the storage modulus, G’, values in the linear viscoelastic (LVE) region. The amount of Ca^++^ influenced the viscoelasticity of the hydrogels; for low levels of Ca^++^ (6 mM), G’ was about 20 Pa, while for medium (8 mM) and increased levels of Ca^++^ (10 mM), G’ was around 50 Pa and 100 Pa, respectively.

Gelatin is a water-soluble protein obtained by the partial hydrolysis of collagen, which breaks the triple-helix structure of collagen into single-stranded molecules [6,7]. It is abundantly used in food, cosmetics, and pharmaceutical products, providing elasticity and structural stability at particular temperatures. Gelatin forms thermoreversible gels once its solutions are cooled below the sol–gel transition temperature. The dispersed molecules in random coils are assembled into ordered triple-chain helices [6,7,8]. Thus, the solution becomes a physical elastic hydrogel. Upon an increase in temperature, the triple-helix conformation returns to a coiled state again, and therefore the gel reversibly melts into a solution [6,7,8]. Besides utilizing mammalian gelatin, a growing interest in fish gelatin has been observed in particular cases since the characteristic phase transition temperatures (sol–gel or gel–sol) are lower, below 10 °C; yet, the concentration threshold to obtain the gels is higher [8].

Over the last decades, several combinations of either polysaccharides or proteins, or even polysaccharides with proteins (including inevitable gelatin), have been utilized to obtain the functionalized mechanical properties of hydrogels [1,2,9,10,11,12,13,14]. Moreover, the mechanical characterization of such hydrocolloid aqueous solutions, and the resulting gels, are studied beyond the LVE, including large-amplitude oscillatory shear (LAOS), among other methods [6,9,10,11,12,13,14]. When investigating binary gelatin-alginate systems for in situ gelation and *ex situ* individual gel layers, Goudoulas and Germann [9] reported comparable storage modulus G’ overshoots for the in situ gelation samples. In contrast, the behavior of modulus G″ loss was very different and was affected by gelatin concentration, aging time, and probing temperature [9]. A common feature of all the studied systems is a highly structured network, mainly because of entangled or crosslinked biopolymers. Generally, the biopolymer concentration is a crucial parameter. For low concentrations, weak gels are observed with progressively increased nonlinear *T* measures in the strain sweep (i.e., validating intracycle shear thickening) but without an apparent G″ overshoot [10]. For relatively high biopolymer concentrations (e.g., above 1 wt.%), the Mw determines both the range of the LVE and the magnitude of the G″ overshoot [11]. When using LAOS analysis of the instantaneous shear stress for concentrated emulsions, Anvari and Joyner (Melito) [12] showed that the nonlinear strain hardening and softening measures are related to the fish gelatin—gum Arabic complexation of the aqueous phase. Most valuably, the quantitative parameters (*I*_3_/*I*_1_) and nonlinear measures (e_3_/e_1_, v_3_/v_1_, S, T) obtained by the LAOS analysis of the shear stress waveform extend the microstructural assessment of the polymeric systems beyond a descriptive approach of a strain sweep [15]. In order to obtain such quantitative parameters for multicomponent hydrogels, researchers propose mechanisms of shear-induced temporary structure formation and reformation based on increased or decreased molecular interactions. For example, the amplification of energy dissipation [16], the shifting of the nonlinear behavior onset and increase in yield stress [17], and the abrupt yielding of the gel structure as a function of the protein fractionation [18] from the LAOS analysis have all been reported.

With increased strain amplitude during LAOS tests, most gels demonstrate a transition from predominantly elastic to plastic and yielding behavior [2]. Recently, the “rheological fingerprint” of soy protein heat-set gels and the hydrolyzed derivatives of soy protein isolate (SPI) were established [19]. The SPI exhibited stronger intracycle shear stiffening at the medium strain range (between 100% and 200%), and the hydrolyzed gels showed stronger intracycle shear thinning because of the smaller peptides. Interestingly, the hydrolyzed gels yielded more gradually than the SPI at the large strain regime [19]. Tian et al. [20] aimed to correlate the rheological fingerprint of pectin aqueous-ethanol gels with the aroma compounds released using small amplitude oscillatory shear (SAOS) and LAOS experiments. They analyzed the Lissajous-Bowditch curves (L-B) at specific strain points and the higher stress harmonics. The results revealed strong positive correlations between the aroma compound concentration and the nonlinear measures v_3_/v_1_ and S/T ratio, suggesting that the nonlinear rheological response affects aroma release [20].

All studies cited above refer to the in situ preparation of hydrogels or a relocation to the rheometer during initial gelation. Although, in particular studies, the supporting images showed nonflowable cohesive gels, these efforts are not performed on individual *ex situ* gel layers. Goudoulas and Germann [9,21] applied a method for systematically preparing *ex situ* gelatin layers and binary gelatin-alginate gel layers with a desired thickness and diameter. Among others, they investigated the LAOS nonlinear behavior for various concentrations of the binary system [9] and different gelation times [21]. The layers showed exclusively intercycle shear-thickening behavior and intracycle strain-stiffening results, indicating these gels had similar microstructures.

The present study aims to mainly investigate the nonlinear LAOS response of alginate-Ca^++^ gel layers prepared individually. To our knowledge, this is the first reported effort with such layers. In order to extend the previous knowledge on the alginate and gelatin gels [5,9,21], we prepared alginate-Ca^++^ and highly concentrated binary layers to achieve comparable G’ and G″ moduli. By analyzing the L-B curves and using LAOS analysis to obtain the intracycle nonlinear measures (e_3_/e_1_, v_3_/v_1_, S, T), the microstructure of these gels was assessed. We intergrade the nonlinear behavior of *ex situ* gel layers by identifying the differences and similarities between the two types of layers.

## 2. Materials and Methods

### 2.1. Materials

In this study, powder of sodium alginate Kimica I-3G (KIMICA Corporation, Osaka, Japan) was utilized to prepare alginate solutions. This alginate is extracted from *Macrocystis pyrifera* brown algae, and the batch was supplemented by a data sheet; the molecular weight (Mw) was 334 kDa and the G/M = 1.67. In addition, we used calcium chloride dihydrate (CaCl_2_∙2H_2_O, 99%) (Alfa Aesar, Karlsruhe, Germany) without further purification to produce calcium solutions at different concentrations. Sodium azide (NaN_3_) (AppliChem GmbH, Darmstadt, Germany) was utilized in the solutions as the biocide. Gelatin type A from porcine skin with an Mw of 50–100 kDa (G2500, Sigma-Aldrich, Munich, Germany), with a pI 8.0 ± 1.0 and 300 Βloom, was also used in this study.

### 2.2. Preparation of Biopolymers Solutions

Each sample preparation started by dissolving sodium azide (NaN_3_) in distilled water. In detail, 250 mL of 0.1 wt.% NaN_3_ was prepared and left stirring for 24 h at room temperature (approximately 25 °C). For preparing alginate solutions, the stock solution of 0.1 wt.% NaN_3_ was utilized with sodium alginate powder and distilled water to achieve the desired material concentrations. Batches of 50 mL and 2 wt.% sodium alginate solution were prepared with slow mechanical stirring for 6 h at 25 °C. When the alginate was fully dissolved, the sample was stored in the refrigerator at 5 °C.

Aqueous solutions of CaCl_2_ were prepared at two different concentrations of 100 and 200 mM and 250 mL volume by dissolving the appropriate amount of CaCl_2_ in distilled water with continuous stirring for 4 h at 25 °C. Although all aqueous solutions were stored in the refrigerator, before each layer preparation, the solution was equilibrated at room temperature (approximately for 1 h).

For the gelatin-alginate layers, gelatin powder was dissolved in the aqueous NaN_3_ solution at 45 °C, and slow mechanical stirring was applied. The heating and stirring conditions were applied for up to 3 h to dissolve the solid gelatin. Typical laboratory glass bottles with screw caps were utilized to prevent evaporation, and their weight was recorded before and after the dissolving stage. The solutions and, consequently, the gel layers were prepared without pH adjustment, i.e., at around 5.6, which was the native pH value of gelatin. The binary mixtures were prepared by mixing equal weights of gelatin and alginate solutions and left under continuous stirring for 4 h at 45 °C. During preliminary tests for several combinations, we found that the 10–10 wt.% gelatin-alginate gel was the one with comparable G’ values to the alginate-Ca^++^, and thus, we used this. In all samples, the NaN_3_ final concentration was estimated as 0.02 wt.%. All liquid batches were stored in the refrigerator, yet, the gelatin-alginate mixture was re-liquefied before any utilization at 45 °C with continuous stirring for 15 min.

### 2.3. Ex Situ Gelation of Individual Layers

**Figure 1:**
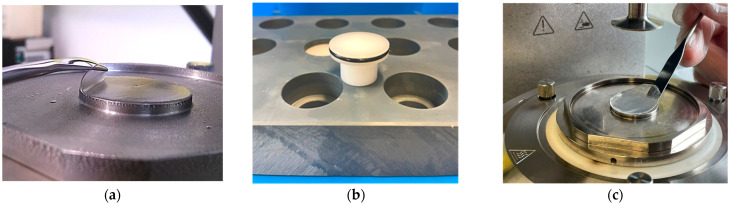
(**a**) Typical binary layer prepared at 5 °C, effectively placed on the lower plate of the rheometer; (**b**) The Teflon stage, having a sealing O-ring, where the alginate-Ca^++^ layers were prepared, along with details of the multiholder PVC base; (**c**) positioning an alginate-Ca^++^ layer on the lower plate of the rheometer.

For the large amplitude oscillatory strain sweep measurement, we prepared individual gel layers in an undisturbed manner and with controlled thickness using available custom-designed casting modules that were designed and utilized previously [9,21]. In brief, for the gelatin layers, the module comprises the main cylindrical metallic body, an inner detachable Teflon stage with a coaxial and reciprocating design that carries the solution sample, and a cover to prevent evaporation. The upper Teflon component diameter was 25 mm, which was identical to the lower base of the rheometer geometry. Typically, an amount of 0.6 mL using a pipette was applied to the Teflon stage to create a gel layer of about 1 mm (thickness). A gentle filling of the casting module ensured a gel layer free of bubbles. To create the layer, the liquid sample was slowly cooled down to room temperature for 1 to 1.5 h, and the sample was then stored in the refrigerator at 5 °C. A representative case is shown in Figure 1a.

We prepared *ex situ* alginate-Ca^++^ layers using a modified casting mold to prepare multiple gel layers, as depicted in Figure 1b. The inner cylindrical component is a Teflon stage with a flattened head with a diameter of 30 mm. The component can easily fit into the PVC base to a desirable height before applying the alginate solution. Using a pipette, we placed 0.9 mL of the Kimica I-3G alginate solution at a concentration of 2 wt.% on the Teflon’s flattened head. On top of that, 0.3 mL of CaCl_2_ solution was added using a pipette for the two concentrations of 100 and 200 mM. To prevent the sample’s evaporation, we placed an identical Teflon head upside-down, sealing the gelling mixture from above. Next, the alginate solution was left to react with the Ca^++^ ions, and the gel sample layer was created overnight at room temperature. Typical alginate-Ca^++^ layer thickness was about 1 ± 0.1 mm. To adjust the layer diameter for the rheometer’s geometry (parallel plate geometry, diameter of 25 mm), we accurately trimmed the periphery using a sharp scalpel on a metal mold with a comparable diameter. Subsequently, we positioned them on the lower geometry, as shown in Figure 1c. Preliminary efforts to utilize the gelatin-alginate casting mold were unsuccessful mainly because of the combination of the low alginate solution viscosity and the overflow when the CaCl_2_ solution was applied. The outcome was notably uneven in the upper surfaces of the layers after the overnight gelation. It seems that the slightly wider diameter of the modified multiholder casting mold allowed us to achieve a flat upper surface, a necessary feature for the probing specimen on the geometry.

Further detailed information, including the chemical structure of the alginate [22], the established ionically gelation steps leading to the so-called “egg-box” formation [23,24], and the assembly scheme of different layers [21], is provided in the Supplementary Material file, Section S1.

### 2.4. Rheological Characterization

We performed the rheological experiments using an MCR 502 rheometer (Anton Paar, Graz, Austria), employing a sand-blasted parallel plate geometry of 25 mm diameter (Figure 1a,c). In all experiments, we enabled the features TrueStrain and TrueGap of the rheometer. We applied the rheometer’s hood and the solvent trap to achieve an enclosed saturated volume. Typical gap values were between 0.8 to 1.1 mm, defined by the thickness of the layers. Awareness of the normal force impact during the gap adjustment had been reported previously, and we followed the proposed protocol [9]. The gel layers experienced normal force Nf values between 5 and 10 N during the gap fine-tuning by the upper plate. A subsequent equilibrium time was around 15 min to overcome any normal force effect, and the measurement started when Nf was less than 0.5 N.

The strain amplitude varied between 0.1 and 1000% strain, and the applied frequency was 1 rad/sec. For the binary gel layers, the temperature was 5 °C, and for the alginate-Ca^++^, it was 25 °C, and the measurement duration was 26 and 22 min, respectively. We performed all rheological measurements in triplicate, and the averaged results are presented here. We observed deviations from the mean values of around 2.5 and 5% in the LVE and LAOS regions, respectively. All instantaneous shear-stress waveform data were analyzed using the MITlaos software (MITlaos Ver. 2.1 Beta).

## 3. Results

### 3.1. Strain Sweeps

**Figure 2:**
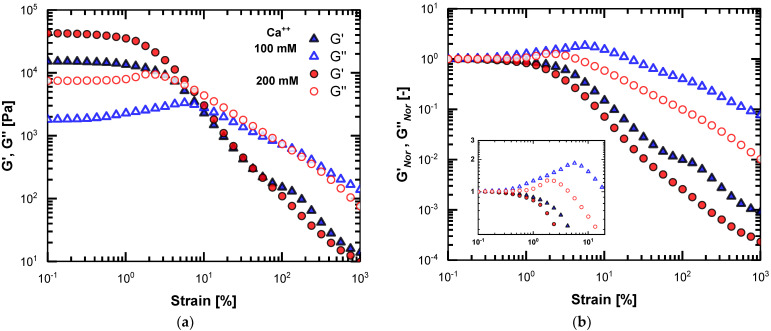
Strain sweeps of the 2 wt.% alginate-Ca^++^ layers for the two studied Ca^++^ concentrations: (**a**) actual strain sweeps data; (**b**) normalized data to the values at 0.1% strain; the inset plot provides details at the shear-thickening onset.

The strain sweeps of the alginate-Ca^++^ layers are shown in Figure 2. The left-hand side of Figure 2 demonstrates the actual LAOS data for the two concentrations, whereas the right-hand side of Figure 2 gives the same data in a nondimensional manner. The storage modulus G’ and loss modulus G″ data were normalized to the values at 0.1% strain. The nondimensional LAOS data were also presented (analogously) in previous studies [10,25,26].

The LVE region is exceptionally narrow in both cases. By considering the systematic differences (of 5%) in the moduli values (for G″ is an increase) as a significant indication of deviation from LVE, we recorded the nonlinear onset at about 0.4% strain for the 100 mM samples and around 1% for the 200 mM samples. In Figure 2a, beyond the crossover of the curves, the moduli converge independently of the calcium concentration. In Figure 2b, no crossover is expected, and the normalized moduli G’*_Nor_* deviate due to different G’ values at 0.1% strain. The behavior of G″*_Nor_* is similar. As the inset shows the details at shear-thickening onset, the increase in G″ overshoot can easily be quantified to 87% for 100 mM (from 1800 to 3320 Pa). Likewise, for 200 mM, the G″ peak is 27% higher than the initial value (from 7450 to 9470 Pa). As reported in Section 2.4, we indicate that in the LAOS region, the standard deviation was close to 5% of the average shear-stress values. This is valid for the current strain-sweep data and, thus, the reported nonlinear measures obtained by the MITlaos analysis.

**Figure 3:**
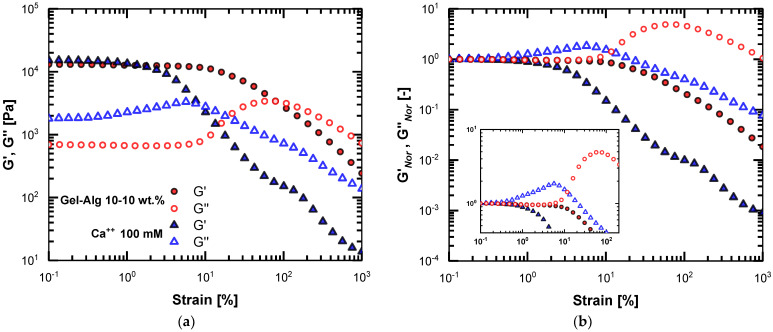
Strain sweeps of the gelatin-alginate layer and 2 wt.% alginate-Ca^++^ layer for comparison: (**a**) actual strain sweeps data; (**b**) normalized data to the values at 0.1% strain; the inset plot provides details at the shear-thickening onset.

In order to compare this with the binary gelatin-alginate layers, we performed similar strain sweeps on the 10–10 wt.% gelatin-alginate layers, as this combination of concentrations gave comparable storage modulus values. The results are given in Figure 3.

We observe an extension of the LVE in the case of the gelatin binary layer. The onset of the nonlinear response occurs at approximately 10% strain, and sharp shear-thickening behavior is apparent, i.e., the G″ overshoot. The above LAOS pattern is well-established for gelatin gels [8,9,21] and for crosslinked polysaccharide hydrogels at high concentrations [11]. The G’ of the two layers are alike, yet, the G″ values differ considerably, indicating a less viscous contribution for the 2 wt.% alginate-Ca^++^ layer. As shown in Figure 3a, the gelatin binary layer retains the elasticity at the medium strain amplitudes. Although the magnitude at the crossover points is comparable (around 3400 Pa), the critical strain is an order of magnitude higher for the binary layer, i.e., about 80%, whereas, for the alginate-Ca^++^ layer, it is 8%. Interestingly, the G″ exhibits a similar rate of decrease as the G″*_Nor_* slopes, as both layers are similar, as shown in Figure 3b. Finally, at the limit of the large amplitudes, both gels are viscous materials, as G″*_Nor_* is two orders of magnitude higher than G’*_Nor_* (depicted in Figure 3b).

### 3.2. 3D Lissajous-Bowditch Curves

A helpful tool to visualize the intracycle shear stress is the 3D Lissajous-Bowditch (L-B) curves. Generally, the L-B curves project the intracycle shear stress waveform either in the viscous or the elastic domain. Therefore, these are closed curves, which at the LVE region should be elliptical as a function of strain (elastic domain) and almost cyclical as a function of shear rate (viscous domain) [2,15].

**Figure 4:**
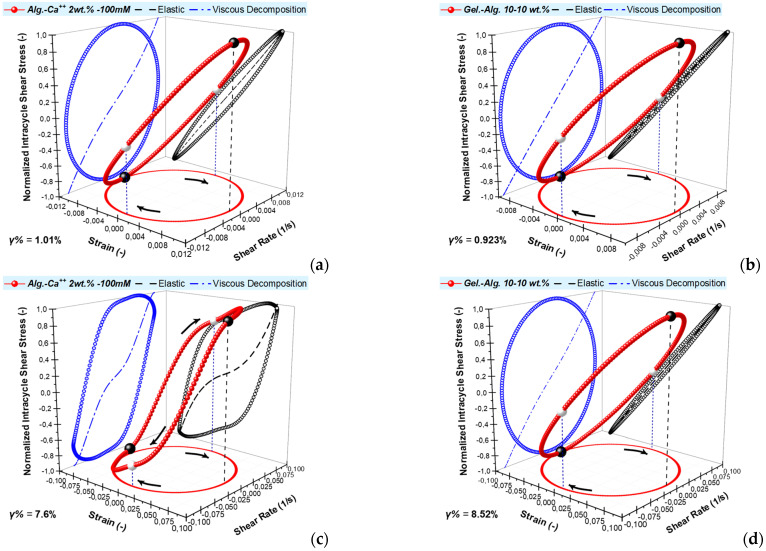

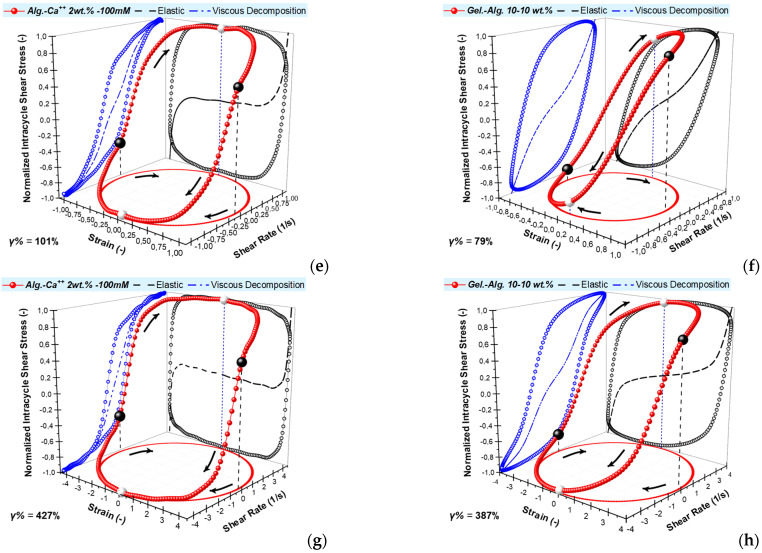
The 3D L-B curves for the alginate-Ca^++^ layer (**a**,**c**,**e**,**g**) and gelatin-alginate layer (**b**,**d**,**f**,**h**). The red-filled points indicate the total intracycle shear stress, and the blue and black points show the viscous and elastic projections, respectively. The noncontinuous lines are the viscous (blue) and elastic (black) stress contributions obtained by the MITlaos software.

The 3D representation of the intracycle stress, as in Figure 4, is suitable for perceiving the decomposed stress in the elastic and viscous domain. All instantaneous intracycle stress data are normalized to the maximum stress of each shear-stress waveform. The abbreviations on the top of the subplots provide info for the compounds of the particular layer. In order to assist with the comprehension of the L-B plots, we applied the red-filled points to the total intracycle shear stress and open blue and black points to the viscous and elastic projections, respectively. The blue dash-dotted line and the black dashed line are the viscous and elastic contributions obtained by the MITlaos software. The enlarged white points indicate the zeroing position of the strain, while the enlarged black refers to the zeroing position of the shear rate, which should be at 90 degrees as the strain and shear rate have an orthogonal correlation [15].

The nonidentical values of the strain amplitude between Figure 4a–d are due to the division of the probing strain range (the slight shift is also traceable in the LVE of Figure 3). We intentionally chose the *γ*% values in Figure 4e–h to indicate similar nonlinear behaviors and matching nondimensional L-B curves. The alginate-Ca^++^ layer shows early nonlinear elastic behavior, as shown in Figure 4a, and at that point, small but noticeable viscous nonlinearities arise. The elastic and viscous contributions are comparable at around 8% strain amplitude. This is almost the crossover point of G’ and G″ in the strain sweep of the alginate-Ca^++^ layer. Eventually, this layer becomes a viscous material with almost zero elastic component contribution, as shown in Figure 4e, and maintains a viscoplastic L-B curve in the elastic domain [2,15,27]. Figure 4g shows that the total stress practically comes from the viscous component, as the blue line extensively merges with the blue points, i.e., viscous projection of total stress.

The data in Figure 4 reveal more similarities than differences between the gel layers, as depicted already in Figure 3. For a strain amplitude of less than 1%, the gelatin-alginate layer is an entirely elastic material, zeroing the total stress at the maximum intracycle shear rates (white points in Figure 4b). Still, at 8% strain, the gelatin-alginate sample is a purely elastic layer. At 80%, the elastic and viscous contributions are comparable, and finally, at around 400%, the gelatin-alginate layer is similar to the alginate-Ca^++^ at 100% strain, i.e., the total stress is viscous at the white points. The layer of 10 wt.% equal gelatin-alginate follows the same evolution for the normalized stress values in the elastic and viscous domain; however, with an order of magnitude in the difference at the critical strain values. Given that the intracycle stress projections are directly related to structure reformation, we assume that the structural evolution in the layers is analogous but with considerably different critical strain values.

### 3.3. Nonlinear Measures Obtained from Shear-Stress Decomposition

Developing powerful tools to effectively and quantitatively describe the nonlinear behavior under LAOS resulted in the shear stress decomposition and the associated nonlinear measures [15,27,28]. In order to list all the nonlinear measures in detail is out of the scope of this study here. Besides, several papers give the whole list and the specifications of every nonlinear measure [2,13,15,21,27,28]. We will restrict ourselves to the measures used to quantify the intracycle nonlinear viscoelasticity regarding the stiffening and the thickening, that is, the stiffening ratio S and the thickening ratio T. The latter (T) is defined by the *η*’*_L_* and *η*’*_M_*, which are the instantaneous viscosities at the highest ($\dot{\gamma }$ → ${\dot{\gamma }}_{0}$) and lowest ($\dot{\gamma }$ → 0) strain rate, respectively. At the same time, the former (S) correlates with the zero-strain modulus G′*_M_* obtained by the differentiation in the shear stress at *γ* → 0 and with the large deformation modulus G′*_L_* defined from the secant value as *γ* → *γ*_0_. The corresponding equations are

S = (G’*_L_* – G’*_M_*)/G’*_L_*, (1)

T = (*η*’*_L_* − *η*’*_M_*)/*η*’*_L_*, (2)

Equations (1) and (2) provide dimensionless indices to quantify a relative amount of strain stiffening (S ratio) and shear thickening (T ratio). According to the related analysis [15,28], when S = 0 and T = 0, this represents a linear viscoelastic response; S > 0 indicates intracycle strain stiffening, and T > 0 represents intracycle shear thickening. As for the negative values, S < 0 corresponds to intracycle strain-softening and T < 0 to intracycle shear-thinning.

**Figure 5:**
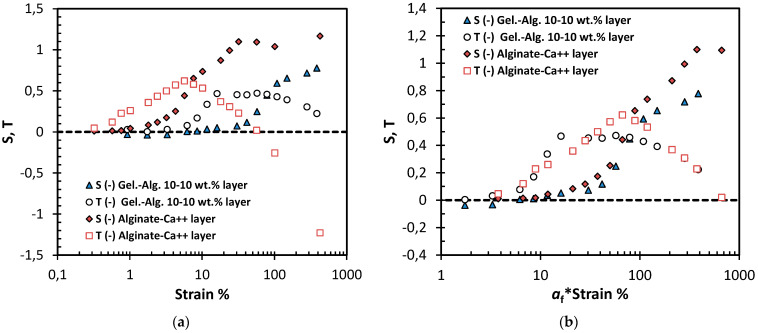
Quantitative analysis of nonlinear response of gelatin-alginate layer and the alginate-Ca^++^ layer: (**a**) the strain-stiffening ratio S, (filled symbols) and shear-thickening ratio T, (open symbols) are given as the functions of the strain amplitudes; (**b**) data from (**a**) rescaled, with a shifting factor *a*_f_ for the strain (equal to 11.7) of the alginate-Ca^++^ layer.

Figure 5a shows the results of the quantitative analysis of the layers’ nonlinear response. The departure from the linear elasticity is observed early for the alginate-Ca^++^ layer, and the nonlinearities are clearly viscous in origin, similarly for the gelatin-alginate layer. The strain-stiffening ratio S for both layers indicates intracycle strain-stiffening, although a minor negative region exists for the gelatin-alginate gel. The rates of increase for the elastic and viscous nonlinearities are similar, given that the elastic nonlinearities appear later than the viscous ones. The T ratio indicates shear-thickening viscous nonlinearities in the beginning and shear-thinning behavior at higher strains, particularly for the alginate-Ca^++^ layer. It has been proposed that shear thickening originates from a temporary network of superstructures/hyper-entanglements [29]. The network has a higher density of junction points at higher concentrations and, thus, longer relaxation times. Consequently, concentrated materials thicken even at lower frequencies [29], as in our case of 1 rad/s. The structural network of the alginate-Ca^++^ layer is based on the propagation of the alginate egg-box dimers. Crosslinks are formed between separate biopolymer chains at the interaction points of Ca^++^ and the alginate’s carboxyl groups. This reduces the electrostatic repulsion between alginate molecules and facilitates the gel structure [24]. The previously described sequence, which results in monocomplexes, dimers, and finally multimers, is terminated to a ‘zipping’ conformation state that is substantially controlled by the hydrogen bonds and the Ca^++^ concentration [24]. We provide a detailed scheme of these steps in the Supplementary Material file (Figure S3). Based on previous kinetics results [5], we believe the crosslinking network of the overnight alginate-Ca^++^ layers reached a steady structural state. The dissociation of crosslinks due to increased deformation is known to cause intercycle strain-softening in hydrogels [26]. Up to 1% strain, no dissociation is expected, and the network is mainly deformed elastically, as shown in Figure 4a. Between 1% and 10% strain amplitude, a gradual dissociation occurs. During this transitional phase of the gel structure, the network (made up of semiflexible bundles) undergoes a rotation of the bundles in the direction of the shear. Next, a transition from a bending-like to a stretching-like deformation is known to cause intracycle strain-stiffening [26]. Subsequently, at large strains (in our case, above 10%), unzipping the egg-box bundles and dissociating the Ca^++^ crosslinks could lead to the observed intercycle strain-softening.

The first overview of Figure 5a shows different behaviors for the two layers, yet, there is an indistinct proportion. Figure 5b shows the data of the alginate-Ca^++^ layer rescaled with a shifting factor *a*_f_ for the strain. Although not identical, the S and T curves show significant similarities between the two types of hydrogels. In particular, the intracycle viscous nonlinearity ratio T showed only a few points that deviated between the two types of layers. Such a shift in the critical strain points reveals a similar structural evolution (or reformation) in both layers due to the imposed large deformations.

## 4. Discussion

The strain amplitude dependency of G′ for the gelatin-alginate is similar to the one obtained for the gels of cod gelatin extracted at different pH levels and at comparable concentrations and probing temperatures (10 wt.%, t = 4 °C) [8]. The critical parameter of the Mw was also in the same range as the present study, i.e., an average Mw of about 130–150 kDa and a wide Mw distribution. On the contrary, the loss modulus G″ had different behavior in our study, showing overshoot. The working hypothesis is that the gel preparation technique, i.e., as individual layers, contributes to achieving a similar bulk microstructure, as was shown previously for gelatin and gelatin-alginate in situ and *ex situ* gels [9,21]. However, when comparing the structure at a molecular binding lever, alginate-Ca^++^ hydrogel differs significantly from a crosslinked system. Nevertheless, in the next paragraph, we will argue on this hypothesis as possibly the bulk microstructures of the two layers are alike.

Alginate and low methoxy pectin (LMP) possess the same ionic crosslinking potential when reacting with Ca^++^ ions. John et al. [26] studied the response of calcium-crosslinked LMP gels under LAOS. The gels exhibit a significant overshoot in the loss modulus G″ and intracycle strain-stiffening, which are characteristics that are related to the degree of egg-box bundling. Some of the results are similar to those presented in Figure 3 and Figure 5, as the strain sweeps and L-B curves for a low-to-moderate Ca^++^ concentration fit with our results. John et al. [26] observed that the nonlinear responses diminish when the bundle radius and the extent of bundling decrease below a critical value. They identified different pectin-Ca^++^ concentration regimes based on the semiflexible/flexible nature of the gel network. Furthermore, it was proposed that the formation of dimers is followed by their lateral aggregation when calcium ions are added to LMP [30]. At low Ca^++^ concentrations, LMP chains aggregate laterally and form egg boxes and their bundles, showing the characteristics of a semiflexible polymer network [26]. As LMP and alginate are similar regarding Ca^++^-induced gelation, we strongly believe a similar structural reformation applies to our study. This is the reason for the comparable strain-sweep pattern to the work of John et al. [26] at low-to-moderate ratios of Ca^++^-to-carboxylic groups (COO^–^), showing a G″ overshoot while the G’ gradually decreases. Moreover, the hierarchical structures of a typical agglomerate species during LMP-Ca^++^ sol–gel transition were proposed recently by Huang et al. [31]. They showed that the bulk structure of LMP-Ca^++^ gels consisted of interior mass-fractal structures of aggregate species in larger formations of agglomerates. They reported the cylindrical LMP-Ca^++^ bundles to a length of 28 nm and a width of 4 nm. They estimated the radius of the aggregates to be equal to 156 ± 4 nm, whereas the radius of the agglomerates R*_g_* was 3200 ± 150 nm [31]. Actually, the characteristic length of 3.2 microns is comparable with the deformation length during a LAOS measurement, where, for example, a 1% strain amplitude creates a deformation of 10 microns for a gap of 1 mm. However, all strain values refer to the rim and the upper plate. Thus, the bulk mass of a gel is subjected to continuously increased deformation lengths, between 0.1 microns to a few millimeters, during a full strain sweep. Similar characteristic lengths apply to gelatin, as the triple helix length (depending on the Mw, looped and nonlooped molecules, etc.) are between 60 to approx. 100 Å [32]. Yet, the subsequent bundles that are formed can reach the scale of microns. Finally, as the gels are created on the hydrophobic Teflon base and to an open-to-air interface, larger agglomerate formations are expected to develop in both types of gel layers. As is evident from the similarities in Figure 5b, the results suggest that such a reason is valid.

## 5. Conclusions

Various chemical and physical gels have been investigated so far using large amplitude oscillatory shear (LAOS) to understand either the physical processes or the reformation of the structure. The gelatin gels are based on thermoreversible gelation. In contrast, the alginate-Ca^++^ layers are created by physicochemical crosslinking. Using shear stress decomposition via MITlaos software, we obtained the elastic and viscous contributions within the nonlinear response of the individual alginate-Ca^++^ layer. The nonlinear response of the alginate-Ca^++^ and gelatin-alginate *ex situ* individual layers were similar, with G″ overshoot. However, the critical strain points were different. Larger-scale structural units (agglomerate/aggregates) are expected to exist in the bulk microstructure, resulting in similar nonlinear patterns. The evolution of this nonlinear behavior is significant from a structural point of view and for practical applications, e.g., food utilization.

## Data Availability

Data contained within the article and Supplementary Materials are available on request from the authors.

## Supplementary Materials

The following supporting information can be downloaded at: https://www.mdpi.com/article/10.3390/polym15061558/s1, Figure S1: Basic monosaccharides, and sample configuration of GG-, MM- and GM- blocks of the alginate molecule; Figure S2: Qualitative scheme of assembling mechanism by Ca^++^ and configuration of alginate molecules for creating the so-called “egg-box” model, along with possible crosslinking conformations; Figure S3: Schematic presentation of microstructural evolution from monocomplexes to multimers of alginate and low methoxy pectin with Ca^++^; Figure S4: Utilization of the individual gelation module for obtaining a gelatin gel layer from the custom-made module.
