# Supplementary material for: Comparative Large Amplitude Oscillatory Shear (LAOS) Study of Ionically and Physically Crosslinked Hydrogels
Source: Polymers (Basel). 2023 Mar 21;15(6):1558. doi: 10.3390/polym15061558 (PMC10051575; doi:10.3390/polym15061558)
Supplement: Supplementary file 1 [file polymers-15-01558-s001.zip › polymers-2272261-supplementary.pdf]

Supplementary Material

Comparative Large Amplitude Oscillatory Shear (LAOS) study of ionically and physically crosslinked hydrogels

Thomas B. Goudoulas [1*], Anna Didonaki [1], Sharadwata Pan [1], Fattahi Ehsan [1] and Thomas Becker [1]

[1] Chair of Brewing and Beverage Technology, TUM School of Life Sciences, Technical University of Munich, Weihenstephaner Steig 20, 85354 Freising, Germany

* Correspondence: thomas.goudoulas@tum.de; Tel.: +49 8161713784

S.1 — Chemical Structure and Assembly Scheme of the Gels

In the following Figures, we provide basic information from acknowledged sources about the chemical structure of the utilized polysaccharide. We show schematic details on the reaction sequence of the alginate molecules with the cations to create the so-called "egg-box" model. We choose a source that extends this info to the low methoxy pectin, as this biopolymer behaves similarly to alginate. Finally, details on the layer's utilization are also presented.

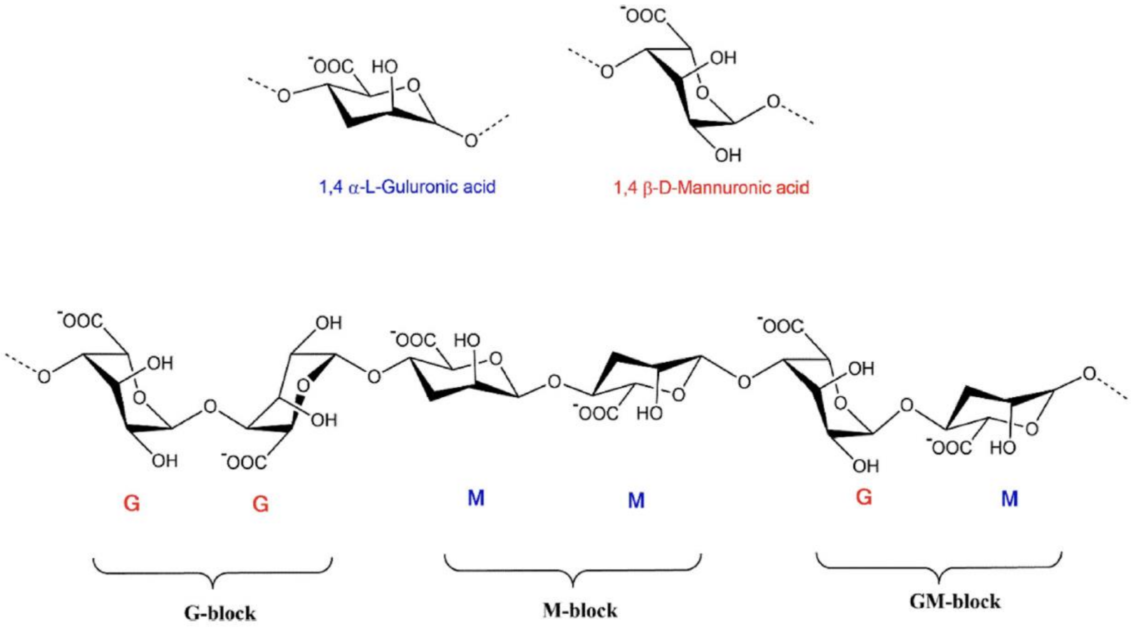

Figure S1. Basic monosaccharides, and sample configuration of GG-, MM- and GM- blocks of the alginate molecule [adapted from Abasalizadeh *et al*. (2020)]

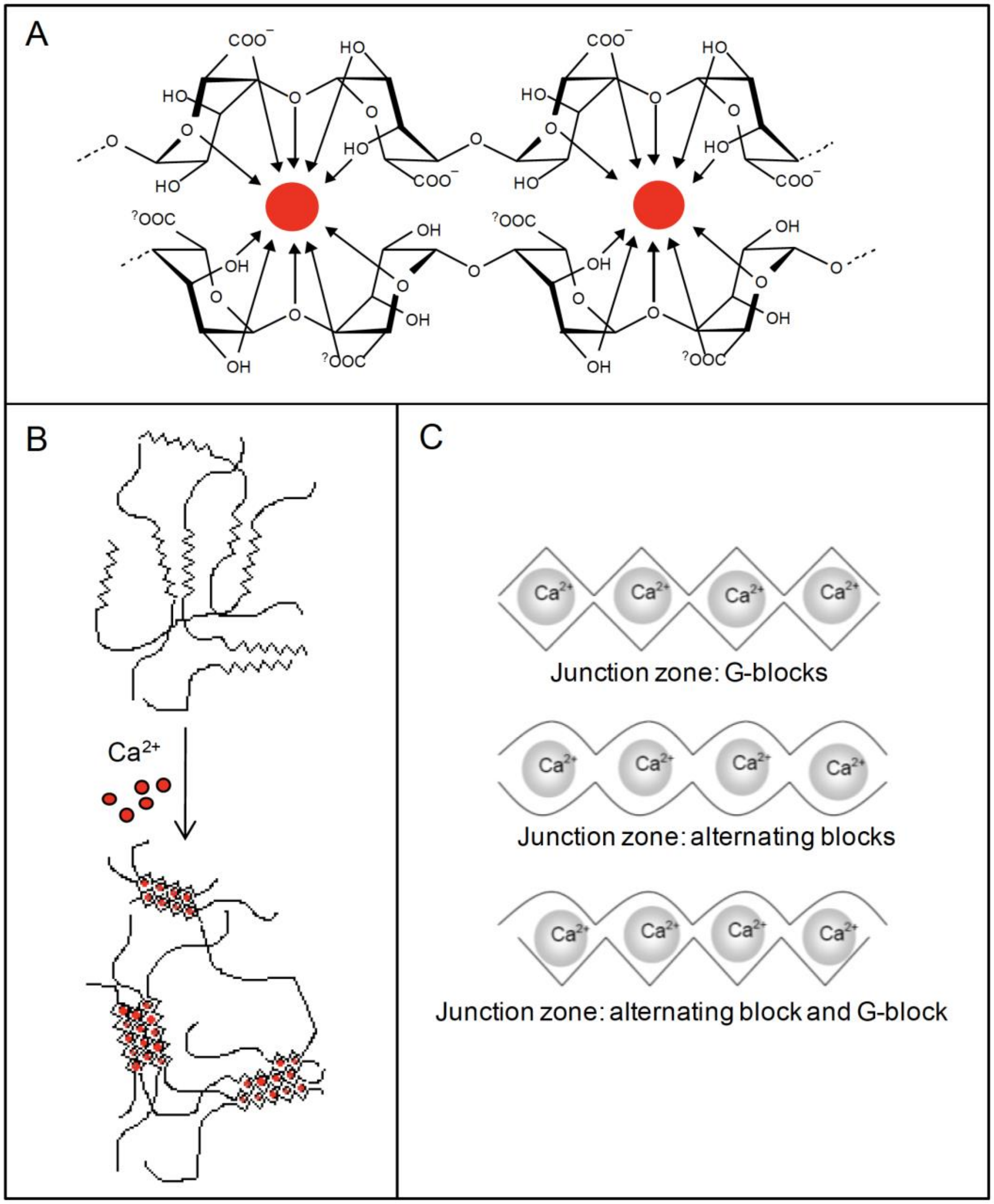

Figure S2. Qualitative scheme of assembling mechanism by Ca^{++} and configuration of alginate molecules. (A) Four consecutive G-residues from two approaching chain segments, creating binding sites for divalent cations (red points); (B) Initiation of an intermolecular network of alginate molecules in the presence of cations, e.g., Ca^{++}, creating the so-called "egg-box" model; (C) Possible crosslinked formations organized in alternative junction zones [adapted from Andersen *et al*. (2015)]

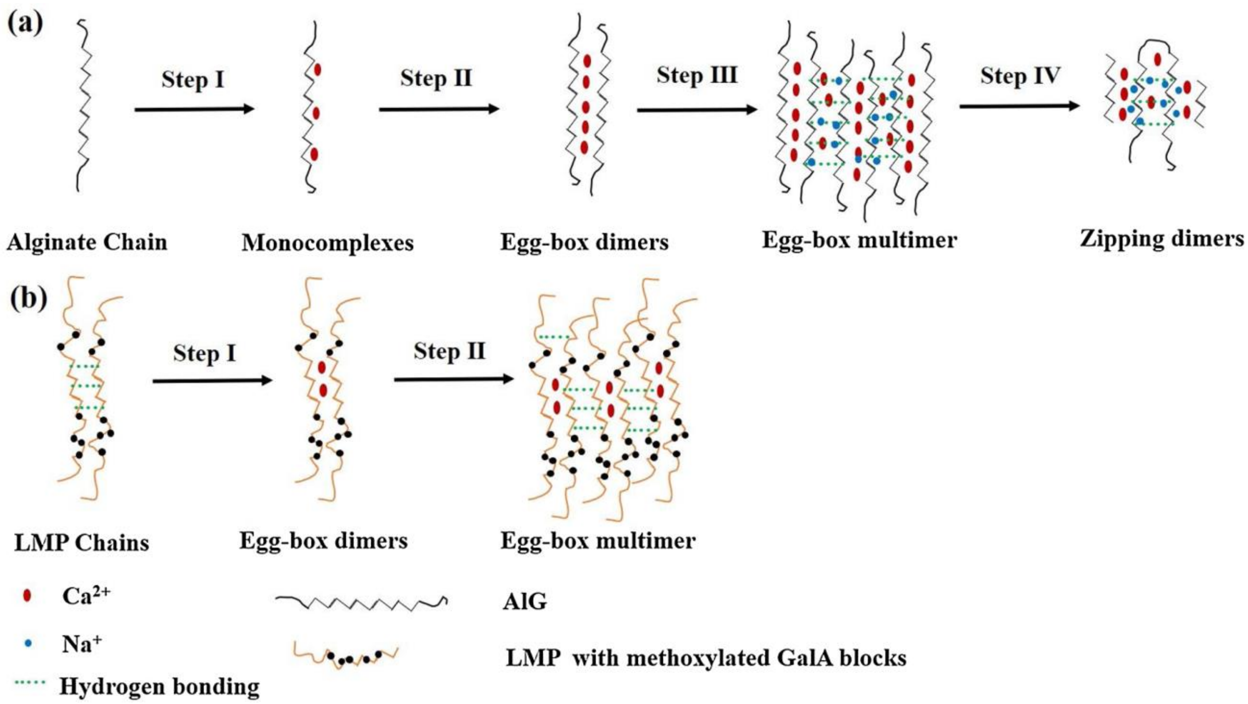

Figure S3. Schematic presentation of microstructural evolution from mono-complexes to multimers, regarding the Ca-binding behaviors to (a) Alginate, (b) Low methoxy pectin. (Abbreviations: ALG: alginate; LMP: low methoxy pectin; GalA: galacturonic acid) [Reprinted from *Carbohydrate Polymers*, 242, Cao *et al*., Copyright (2020), with permission from Elsevier]

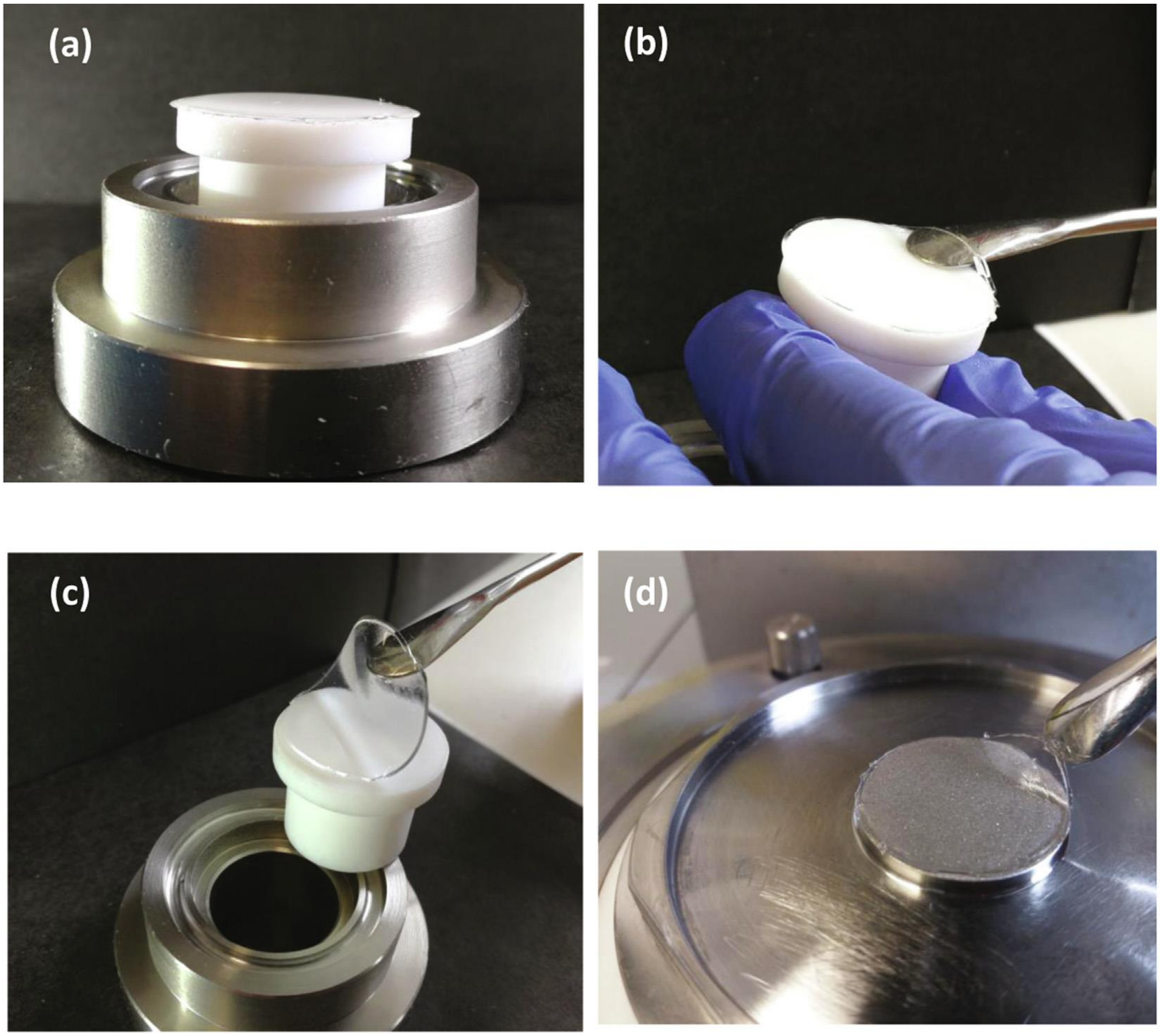

Figure S4. Utilization of the individual gelation module for obtaining a soft native gelatin gel. (a) Custom-made casting module with the prepared individual gel layer. The inner cylindrical Teflon element is raised out of the metallic body; (b) Separation of the gel from the Teflon element; (c) Fully detached layer; (d) Placement on the lower rheometer plate. [Reprinted from *Journal of Colloid and Interface Science*, 553, Goudoulas & Germann, Copyright (2019), with permission from Elsevier]

S.2 — References

Abasalizadeh, F.; Moghaddam, S.V.; Alizadeh, E. *et al*. "Alginate-based hydrogels as drug delivery vehicles in cancer treatment and their applications in wound dressing and 3D bioprinting." *Journal of Biological Engineering* **2020**, 14, 8. https://doi.org/10.1186/s13036-020-0227-7

Andersen, T.; Auk-Emblem, P.; Dornish, M. "3D Cell Culture in Alginate Hydrogels." *Microarrays* **2015**, *4*, 133–161. https://doi.org/10.3390/microarrays4020133

Cao, L.; Lu, W.; Mata, A.; Nishinari, K.; Fang, Y. "Egg-box model-based gelation of alginate and pectin: A review." *Carbohydrate Polymers* **2020**, *242*, 116389. https://doi.org/10.1016/j.carbpol.2020.116389

Goudoulas, T.B.; Germann, N. "Nonlinear rheological behavior of gelatin gels: *In situ* gels and individual layers." *Journal of Colloid and Interface Science* **2019**, *553*, 746–757. https://doi.org/10.1016/j.jcis.2019.06.060
